# Signalling pathways and mechanistic cues highlighted by transcriptomic analysis of primordial, primary, and secondary ovarian follicles in domestic cat

**DOI:** 10.1038/s41598-021-82051-4

**Published:** 2021-01-29

**Authors:** Shauna Kehoe, Katarina Jewgenow, Paul R. Johnston, Susan Mbedi, Beate C. Braun

**Affiliations:** 1 Reproduction Biology Department, Leibniz Institute for Zoo and Wildlife Research, Alfred-Kowalke-Straße 17, 10315 Berlin, Germany; 2 Berlin Center for Genomics in Biodiversity Research BeGenDiv, Königin-Luise-Straße 6-8, D-14195 Berlin, Germany; 3 Leibniz-Institute of Freshwater Ecology and Inland Fisheries, Müggelseedamm 310, 12587 Berlin, Germany; 4 Freie Universität Berlin, Institut für Biologie, Königin-Luise-Straße 1-3, 14195 Berlin, Germany; 5 Museum für Naturkunde, Invalidenstraße 43, 10115 Berlin, Germany

**Keywords:** RNA, Cell signalling, Development, Gene expression, Gene regulation, Sequencing, Transcriptomics, Bioinformatics, Gene expression analysis, Sequencing, Cell biology

## Abstract

In vitro growth (IVG) of dormant primordial ovarian follicles aims to produce mature competent oocytes for assisted reproduction. Success is dependent on optimal in vitro conditions complemented with an understanding of oocyte and ovarian follicle development in vivo. Complete IVG has not been achieved in any other mammalian species besides mice. Furthermore, ovarian folliculogenesis remains sparsely understood overall. Here, gene expression patterns were characterised by RNA-sequencing in primordial (PrF), primary (PF), and secondary (SF) ovarian follicles from *Felis catus* (domestic cat) ovaries. Two major transitions were investigated: PrF-PF and PF-SF. Transcriptional analysis revealed a higher proportion in gene expression changes during the PrF-PF transition. Key influencing factors during this transition included the interaction between the extracellular matrix (ECM) and matrix metalloproteinase (MMPs) along with nuclear components such as, histone HIST1H1T (H1.6). Conserved signalling factors and expression patterns previously described during mammalian ovarian folliculogenesis were observed. Species-specific features during domestic cat ovarian folliculogenesis were also found. The signalling pathway terms “PI3K-Akt”, “transforming growth factor-β receptor”, “ErbB”, and “HIF-1” from the functional annotation analysis were studied. Some results highlighted mechanistic cues potentially involved in PrF development in the domestic cat. Overall, this study provides an insight into regulatory factors and pathways during preantral ovarian folliculogenesis in domestic cat.

## Introduction

Artificial reproductive technology (ART) such as, germ cell cryopreservation and in vitro embryo production can potentially contribute to species conservation when populations decline to a critically small size^1^. However, in practice, the inadequate number of oocytes that are acquired from later ovarian follicle stages limits current applications within endangered species conservation breeding programs^2^. First and foremost, improving female gamete yield is essential in order to progress. Studies involved in optimising these techniques could potentially be applied to human oncofertility and fertility preservation in the future also^3^.

Within the ovary, an oocyte is found inside an ovarian follicle^4^. Ovarian follicles from various developmental stages are contemporaneously present within adult animal ovaries beginning from PrF, PF, SF, to antral follicular stages. Each follicle stage is characterised by cell-specific morphological and physiological features of the oocyte and the surrounding follicular cells^4^. The activation, growth, and development of an ovarian follicle, a process termed ovarian folliculogenesis, is finely regulated by cell-to-cell interactions and coordinated gene expression in the oocyte and surrounding granulosa cells (GCs)^4^. Oocyte recovery for in vitro applications can be achieved by isolating granulosa-oocyte-complexes from later antral follicles however, the majority of oocytes (≥ 90%) are encapsulated in the earliest, dormant PrFs^5^. Therefore, harnessing oocytes from this larger resource could potentially overcome current limitations in the field^6^. Nevertheless, the recapitulation of female germ cell development from the early preantral stage is challenging and remains in its rudimentary stage for mammalian species.

At present, complete oogenesis originating from primordial germ cells has been achieved in mice^7,8^. For other species such as, ovine and feline, more advanced ovarian follicle stages have developed successfully in culture^9,10^. Embryo production from oocytes derived from advanced preantral follicles developed by IVG has been achieved in porcine, buffalo, ovine, and goat but inconsistently so^11^. Evidently, inadequate oocyte maturation rates are especially accountable for this which demands considerable research. Overall, the key to improving the success rate seems to lie within the sequential provision of essential nutrients and growth factors to the follicles based on a profound knowledge on effective biomarkers for the particular ovarian follicular stage^12^. Thus, RNA-sequencing transcriptomic analysis could address fundamental questions surrounding ovarian folliculogenesis and potentially identify genes and biological processes useful for developing IVG methods.

Mainly, elucidating molecular mechanisms during early folliculogenesis has focused on ovarian follicle gene expression in transgenic murine models^13–15^. Transcriptomic profiling from laser capture microdissection (LCM) of ovine GCs and oocytes has been performed along with gene expression profiling at a spatio-temporal level in the same species^16,17^. In bovine, transcriptome analysis of GCs from ovarian follicles has characterised differential gene expression and has investigated gene expression during development of antral follicles^18,19^. Transcriptomic profiling of human oocytes from PrFs and PFs isolated by LCM has revealed putative signalling factors associated with the maintenance and activation of PrF dormancy^20^. Many studies in domestic animals and rodents are based on early ovarian follicle samples obtained from juveniles (fetal or new born). This allows an exact determination of major follicular stages during ontogenesis. In comparison to this, investigating ovarian folliculogenesis in the adult mammalian ovary can provide insight into the mechanisms involved in maintenance of the ovarian reserve and subsequently activation and developmental processes.

Overall, the aim of this study was to elucidate differential gene expression and biomolecular mechanisms in early preantral follicles from the domestic cat using RNA-sequencing data. To do so, PrFs, PFs, and SFs were isolated from domestic cat ovaries utilising a previously established ovarian follicle isolation method^21^. Two major developmental transitions were explored: PrF-PF and PF-SF. Distinct differences in gene expression levels between these two transitions were found. Functional annotation clustering analysis described biological processes (BPs), cellular components (CCs), and molecular functions (MFs) associated with preantral ovarian folliculogenesis in the domestic cat.

## Materials and methods

All chemicals and materials were purchased from Merck KGaA, Darmstadt, Germany unless stated otherwise.

### Sample collection

**Figure 1:**
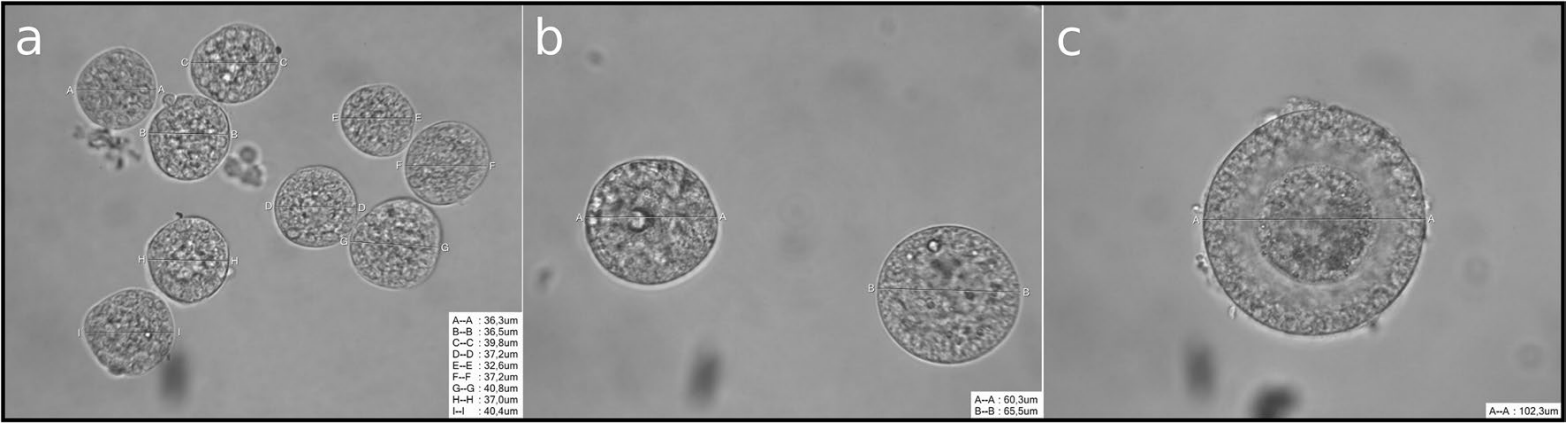
Microscope images of domestic cat preantral ovarian follicles. **(a)** PrF (≤ 45 µm), **(b)** PF (55–70 µm), **(c)** SF (85–110 µm with a visible zona pellucida).

Ovaries were obtained from domestic cats after ovariectomy performed at animal shelters in Berlin. After excision, ovaries were stored in HEPES-MEM medium, supplemented with 3 g/L BSA and 1 × Antibiotic Antimycotic Solution in 50 mL tubes (Sarstedt AG & Co. KG, Nümbrecht Germany) at 4 °C and were processed within a 2–3 h time-frame. The collection of preantral ovarian follicles from domestic cat ovaries has been described previously^21^. In brief, ovaries were pressed through a cell dissociation sieve (60 mesh) in a Dulbecco’s phosphate-buffered saline solution supplemented with BSA (DPBS-BSA—0.3 mg/mL) into a petri dish (Thermo Fisher Scientific, Dreieich, Germany). This cell suspension was pipetted through a series of nylon sieve 40 µm, 70 µm, and 100 µm cell strainers (Falcon, Becton Dickinson Labware, Franklin Lakes, NJ). Flushing the 40 µm, 70 µm, and 100 µm sieves with 6 mL DPBS-BSA allowed for the enrichment of PrFs, PFs, and SFs, respectively. It should be noted that in each flushed sieve suspension, ovarian follicles from other stages could be found. Calibrated and siliconised glass pipettes (coated with Sigmacote) were utilised to collect ovarian follicles. Collected ovarian follicles were measured with an inverse microscope with a 40 x objective (Axiovert 100, Jenoptic, Jena, Germany) equipped with an RI camera and software system (CooperSurgical Fertility and Genomic Solutions, Germany) and ovarian follicle types were determined based on the following measured diameters: PrF ≤ 45 µm, PF 55–70 µm, and SF 85–110 µm, respectively (Fig. 1). Regarding SFs, only those exhibiting a visible zona pellucida were selected for sampling (Fig. 1c). Collected samples were lysed in 10 µL Lysis Buffer 1 from the NucleoSpin RNA Plus XS kit (Macherey–Nagel GmbH & Co. KG, Berlin, Germany) which immediately inactivated RNases. The samples were snap-frozen in liquid nitrogen and stored at − 80 °C until RNA extraction. Sample collection for RNA-sequencing included three biological replicates of pooled follicle samples: PrF (n = 180 follicles from 3 individuals collectively (n = 60 follicles each), N = 3 samples), PF (n = 45 follicles from 3 individuals collectively (n = 15 follicles each), N = 3 samples), and SF (n = 9 follicles from 3 individuals collectively (n = 3 follicles each), N = 3 samples), giving a total of 9 samples. For quantitative real-time PCR (qRT-PCR), additional pooled follicle samples were collected, measured, and stored accordingly: PrF (n = 180 follicles, N = 4 samples), PF (n = 45 follicles, N = 4 samples), and SF (n = 9 follicles, N = 4 samples).

### RNA-sequencing

Total RNA was extracted with NucleoSpin RNA Plus XS (Macherey–Nagel GmbH & Co) from the 9 samples following the manufacturer’s instructions. Total RNA sample integrity and concentration was measured by Agilent High Sensitivity RNA ScreenTape Assay (Agilent 2200 TapeStation system, Agilent Technologies, Inc., Santa Clara, US). Sample concentrations for quantification were determined by Qubit 2.0 Fluorometer (Thermo Fisher Scientific Inc., Waltham, USA). During extraction, genomic DNA was removed by gDNase during on-column DNA digestion. The purified RNA was dissolved in 20 µL RNase-free water and stored at − 80 °C. The cDNA was generated and amplified in 9 cycles with the SMART-Seq v4 Ultra Low Input RNA Kit for Sequencing (Takara Bio, Inc. Mountain View, USA). Libraries were generated (Nextera XT DNA Library Prep Kit, Illumina, San Diego, USA) and sample quality was checked by Agilent 2100 Bioanalyzer (Agilent, Santa Clara, USA) and by Agilent High Sensitivity D1000 ScreenTape Assay (Agilent 2200 TapeStation system). Custom index primers were tagged to the libraries to allow for multiplexing during RNA-sequencing. Libraries were quantified, normalised based on measurements determined by Qubit 2.0 Fluorometer, and sequenced on the Illumina NextSeq 500 system (150 cycles). The raw RNA-Seq data was deposited and released with the BioProject accession number of PRJNA635095.

### RNA-sequencing analysis

The analysis was performed in R (https://www.r-project.org/), R 3.4.4^22^, with Bioconductor (https://www.bioconductor.org/) packages. The analysis workflow, scripts, and a list of acquired packages along with citations are available in the Github repository: https://github.com/kshauna/OvarianFollicleTranscriptomics-DomesticCat.

Raw data consisted of paired-end, double-indexed cDNA library sequencing reads. The PhiX adapter-ligated control was sequenced at a standard concentration of 5%. The de-multiplexed data received in fastq file format was quality checked with FastQC^23^ and summarised with MultiQC^24^. Salmon^25^ quantified transcripts from a FASTA file containing the reference transcriptome and FASTQ files containing the sequence reads. Transcript abundance, counts, and length were summarised by tximport^26^. The DESeqDataSet under three factor levels “type” determined differential gene expression for PrFs, PFs, and SFs with DESeq2^27^. Ovarian follicle type contrasts, PrF versus PF (PrF-PF) and PF versus SF (PF-SF), were designed. Differentially expressed genes (DEGs) were estimated from the un-normalised, paired-end fragments by the Independent Hypothesis Weighting^28^ method with an alpha = 0.05, an adjusted *P* value < 0.05, and absolute log^2^ fold-change of 1 (Supplementary Data S1). For quality control, the log fold-change shrinkage estimates of normalised data were visualised with heatmaps of Euclidean distances and with a principal component analysis (PCA). To assign Entrez gene identifiers (IDs) BioMart^29,30^ was employed. The Entrez IDs were input into the web-based portal “the database for annotation, visualisation and integrated discovery” (DAVID) Bioinformatics Resources 6.8 (https://david.ncifcrf.gov/)^31,32^. The tool DAVID was chosen to: (1) identify major gene groups (functional classification); (2) to elucidate enriched annotation terms (functional annotation chart and clustering, respectively); (3) and to provide an overview of gene annotations (functional annotation table)^31^. Importantly, the last update, DAVID 6.8, occurred in October 2016 and the data analysed here was submitted to DAVID 6.8 at the beginning of 2019. Researchers interested in enrichment analysis should consider a combination of other tools such as gProfiler^33^, Enrichr^34,35^, and/or Metascape^36,37^. However, these portals and others such as, PANTHER^38^, InterMine^39,40^, and GeneTrail2^41^ may be relying on old knowledge bases^37^. Here, the functional annotation and enrichment analysis workflow is summarised as follows: analysis with DAVID 6.8 output gene ontology (GO)^42^ and KEGG (Kyoto Encyclopedia of Genes and Genomes) orthology (KO)^43^ terms which were categorised into functional annotation clusters. The functional annotation tool (FAT) was applied to the GO categories BP, CC, and MF which filtered out broad GO terms based on measured specificity. Default categories were unselected and “GOTERM_BP_FAT”, “GOTERM_CC_FAT”, “GOTERM_MF_FAT”, and “KEGG_PATHWAY” were selected. An over-representative hypergeometric test on the resulting GO and KO terms was performed and the clustering options within DAVID were modified as follows: Similarity Term Overlap 3, Similarity Threshold 0.60, Initial Group Membership 3, Final Group Membership 3, Multiple Linkage Threshold 0.50, and an Enrichment Threshold EASE 0.2. The GO and KO terms with an arbitrary enrichment score (ES) > 0.1 were considered for further investigation. A false discovery rate (FDR), *Q* value (*Q*), corrected for multiple testing of *P* values of the enriched terms. A *Q* < 0.05 was considered as significant. For visualisation purposes the web-based portal Metascape (http://metascape.org) was utilised^36^. Metascape currently does not support the domestic cat but it does support human. As gene annotation databases are primarily compiled for human genes it is useful to use human orthologs for model organisms prior to analysis^36^. As a model organism, the domestic cat shares several female germ cell features to humans: (1) the oocyte proper and the germinal vesicle diameter is equivalent; (2) oocytes reach metaphase II (MII) stage of meiosis after 24 h in culture; and (3) the nuclear configuration is comparable^44,45^. Thus, g:Profiler g:Orth orthology (https://biit.cs.ut.ee/gprofiler/orth)^33^ converted the *Felis catus* Entrez IDs into *Homo sapiens* Ensembl IDs (an orthologous species recognisable by Metascape). The “Express Analysis” in Metascape was selected which automatically removed ontology terms that did not satisfy the minimal statistical criteria. The analysis report produced functional annotation network cluster graphs and ontology enrichment bar plots which were summarised in an Excel workbook, PowerPoint presentation, and Zip folder format. Metascape performed an additional gene annotation, membership search, and enrichment clustering on the gene list. Together, the multi-test corrected *Q* values (DAVID) and *P* values (Metascape) were considered when ranking the enriched terms. The DESeq2 function “plotCounts” plot RNA-sequencing normalised count data of selected genes on a log scale for diagnostic purposes only. For comparison to qRT-PCR results, normalised counts were fit with a negative binomial general linearised model and coefficients and intervals were extracted.

### Gene expression by qRT-PCR

Total RNA extraction for qRT-PCR was performed as described previously. Reverse transcription of RNA into cDNA was performed as described by Hryciuk et al.^46^. Intron-spanning primers were designed according to gene sequences listed in GenBank (Supplementary Table S1). The cDNA was diluted 1:10 and analysed with the CFX96 Real-Time PCR detection system utilising the SsoFast EvaGreen Supermix (Bio-Rad Laboratories GmbH, Munich), at 98 °C and 8 s at different annealing temperatures. Quantification of qRT-PCR products was performed with CFX Manager Software 3.1 (Bio-Rad Laboratories GmbH). Serial dilutions of plasmid DNA carrying genes of interest sequences or of qRT-PCR products were utilised for calibration with β-actin (*BACT*) as a reference gene^47^. Statistical analysis was performed in R using R 3.4.4^22^. The Kruskal–Wallis rank sum test determined *P* values of gene expression. The Wilcoxon rank sum test determined post-hoc pairwise comparisons (*P* value adjustment: Benjamini–Hochberg). Sigma-Plot 10.0 (Systat Software Inc., San Jose, CA, USA) allowed for the visualisation of qRT-PCR statistical results through box plots.

### Ethics declarations

Domestic cat ovaries were obtained from animal shelters in Berlin after routine ovariectomy for the purpose of permanent contraception primarily from stray domestic cat individuals. The surgical procedures were not related to the purpose of the experiment. The animal shelter agreed to donate the excised ovaries which we utilised as samples. Castrations are compliant with the “Protection of Animals Act” in Germany; no further guidelines had to be considered.

## Results

### RNA-sequencing

Raw data, 35 Gb, was obtained after Illumina NextSeq sequencing. FastQC analysis estimated the total sequence distribution between samples (Supplementary Fig. S1a). Sequence length ranged from 59.4 to 76 base pairs (bp) with a guanine-cytosine content of 48.94% on average. Fragment lengths were distributed at approximately 300 bp. The percentage of mapped reads (% aligned) and mapped reads (millions) (M aligned) was estimated with MultiQC (Supplementary Fig. S1a). Trimming of reads was not performed. Log count distributions between samples before and after normalisation were visualised (Supplementary Fig. S1b). The PCA of the nine samples explained sample variance by the first two principal components (PC1 versus PC2) which accounted for 37% and 16% variation, respectively whereby, PC1 separated PrF from PF and SF, whereas PC2 separated PF from SF. This emphasised the sample-to-sample relationships based on ovarian follicle type (Supplementary Fig. S2a). The PC1 versus PC3 for all genes accounted for 37% and 12% variation, respectively (Supplementary Fig. S2b). The inclusion of PC3 explained a reduced proportion of variation between the PF and SF samples. Collectively, the first three PCs explained approximately 65% of total variation. The PC1 versus PC2 for 500 genes accounted for 39% and 19% variation, respectively (Supplementary Fig. S2c). A hierarchical heatmap demonstrated that samples clustered together based on ovarian follicle type (Supplementary Fig. S3). Clustering of all PrF samples was identified with a high degree of correlation. An outlier PF sample was observed (sample_4_S4) but the remaining two samples were closely associated. All SF samples were highly correlated. Overall, the degree of correlation demonstrated a relationship between PF and SF follicle samples. These samples were either less correlated or not correlated at all with the PrF samples (Supplementary Fig. S3). Additionally, sample-to-sample relationships were visualised with hierarchical cluster dendograms (Supplementary Fig. S4).

### Differential gene expression analysis

**Figure 2:**
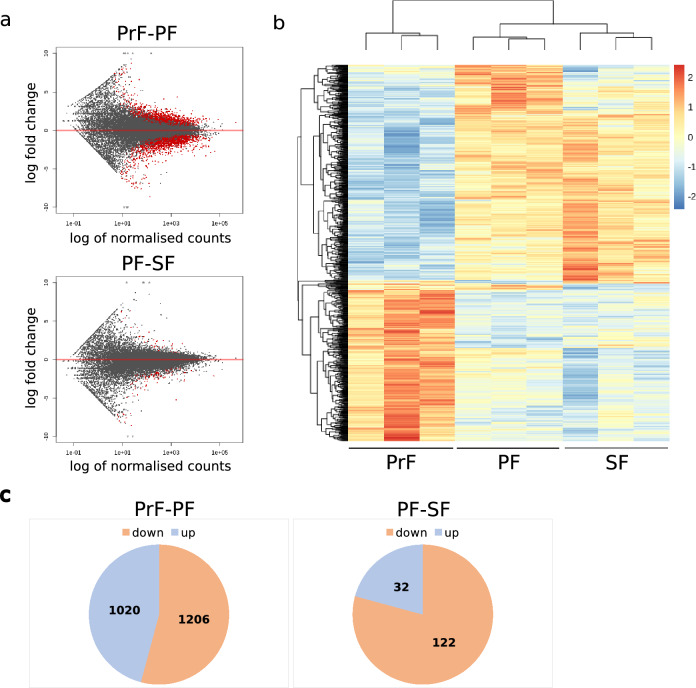
Differential gene expression during preantral ovarian folliculogenesis in domestic cat. **(a)** The estimated number of DEGs between ovarian follicle contrasts: primordial versus primary and primary versus secondary with an adjusted *P* value of < 0.05 and log^2^ fold-change ≥ 1 were visualised utilising MA plots whereby, non-significant genes (values not satisfying the *P* value and log^2^ fold-change threshold) are shown in black and significant genes (values satisfying the *P* value and log^2^ fold-change threshold) are shown in red, log^2^ fold-change is mapped to *y* and the normalised mean is mapped to *x* (transformed to log^10^ scale); **(b)** Patterns of gene expression changes are shown on a heatmap with an adjusted *P* value of < 0.01 and log^2^ fold-change ≥ 1. The colour red represents relative increase in expression, blue represents relative decrease, and pale orange (in the middle of the colour scale) represents no change. The columns are labelled with follicle type, respectively; **(c)** Pie charts summarise the estimated number of DEGs between the primordial versus primary and primary versus secondary contrasts with an adjusted *P* value of < 0.05 and log^2^ fold-change ≥ 1.

The highest number of DEGs was identified during the PrF-PF transition. This included 2,226 DEGs comprised of 1,206 down-regulated and 1,020 up-regulated genes. The lowest number of DEGs was found in the PF-SF transition whereby, the total 154 DEGs estimate included 122 down-regulated and 32 up-regulated genes (Fig. 2a, c). This revealed that the number of DEGs decreased when comparing the PrF-PF and PF-SF transitions. The patterns of gene expression changes across all samples were graphically depicted in heatmap format (Fig. 2b) and summarised with a diagram of ovarian follicle DEGs (Fig. 2c). All differential gene expression lists were combined into one DEG database (Supplementary Data S1). Collectively, 2,380 DEG transcripts including 1,328 down-regulated and 1,052 up-regulated genes were detected in early preantral ovarian follicles in domestic cat.

### Functional annotation clustering analysis

To focus the study, the following aims were established for analysis of the domestic cat data: (1) identify signalling pathways conserved in mammalian ovarian folliculogenesis; (2) select signalling pathways that are most functionally annotated/enriched; (3) extract DESeq2 DEG values for the genes associated with the respective pathways and plot transcript expression patterns for some representatives of it. The main conserved pathways during ovarian folliculogenesis in mammals include: adenylate cyclase; mitogen-activated protein kinase/extracellular signal-regulated kinase (MAPK/Erk); phosphoinositide-3-kinase/protein kinase B (PI3K-Akt); phospholipase C; janus kinase/signal transducers and activators of transcription (JAKS/STATS); SMAD (*Caenorhabditis elegans Sma* gene and *Drosophila Mad*, Mothers against decapentaplegic); and nuclear receptors^48^. Additionally, any over-represented BPs not explicitly defined as “signalling pathway” were taken into consideration.

From 2,226 DEGs within the PrF-PF transition 1,916 DAVID IDs (an internal gene ID defining a unique gene cluster from a single gene entry) were identified resulting in 137 functionally annotated clusters (Supplementary Data S2). Significantly enriched terms included “extracellular region part” (*Q* = 2.10E−07), “focal adhesion” (*Q* = 5.30E−05), “movement of cell or subcellular component” (*Q* = 6.70E−04), and “cell morphogenesis” (*Q* = 3.80E−03). The KO terms such as, “ECM receptor interaction” (*Q* = 1.60E−06) and “focal adhesion” (*Q* = 1.80E−03) were significantly enriched also. Other enriched terms and pathways were involved in “cellular response to growth factor stimulus” and the “phosphatidylinositol 3-kinase (PI3K)-Akt/Protein Kinase B (Akt)” and “transmembrane receptor protein serine/threonine kinase” signalling pathways (Table 1). Additionally, “platelet derived growth factor”, “integrin-mediated”, and “transforming growth factor (TGF)-β” signalling were observed during the PrF-PF transition (Supplementary Data S3). Interestingly, “axonemal dynein complex assembly” was identified in Annotation Cluster 32 and was coupled with “ciliary plasm” and “axoneme cellular components” (Supplementary Data S2).

**Table 1:** Functional annotation clusters identified during the PrF-PF transition in domestic cat using DAVID.

Cluster	ES^1^	Description (DAIVD ID*)	Count^2^	FC^3^	BH^4^	FDR^5^
1	8.47	Extracellular region part	427	1.3	1.1E−7	2.1E−7
2	6.01	ECM-receptor interaction	31	3.4	3.3E−7	1.6E−6
		Focal adhesion	49	2.3	5.6E−6	5.3E−5
		PI3K-Akt signalling pathway	49	1.4	2.1E−1	2.2E1
3	5.23	Focal adhesion	64	1.9	9.9E−5	1.8E−3
4	5.01	Dilated cardiomyopathy	25	3.0	1.3E−4	1.8E−3
5	4.83	Endopeptidase inhibitor activity	31	2.5	3.9E−3	4.6E−3
6	4.29	Collagen metabolic process	14	3.5	2.8E−2	1.5E−1
7	4.01	Movement of cell or subcellular component	182	1.4	1.2E−3	6.7E−4
8	3.68	Glycosaminoglycan binding	27	2.1	4.9E−2	5.9E−1
9	3.07	Epithelium development	115	1.4	2.2E−2	1.0E−1
10	2.94	Cellular response to growth factor stimulus	62	1.5	8.7E−2	9.7E−1
		Transmembrane receptor protein serine/threonine kinase signalling pathway	38	1.5	4.4E−1	2.2E
11	2.91	Meiotic cell cycle	36	2.2	8.8E−3	2.4E−2
12	2.7	Meiosis I	20	2.5	6.1E−2	5.8E−1
13	2.62	Embryonic morphogenesis	75	1.5	4.3E−2	3.8E−1
14	2.6	Cellular response to amino acid stimulus	15	3.1	3.7E−2	2.8E−1
15	2.59	Cell morphogenesis	146	1.5	3.4E−3	3.8E−3
16	2.56	Negative regulation of cellular component movement	34	1.7	1.6E−1	3.1E0
17	2.56	ECM structural constituent	12	2.8	2.0E−1	3.6E0
18	2.48	Vasculature development	70	1.5	1.2E−1	1.7E0
19	2.38	Cilium organization	36	1.7	1.6E−1	2.7E0
20	2.38	Response to oxygen-containing compound	106	1.4	1.2E−1	1.6E0

The top 20 categories grouped by similar GO and KO terms are listed. The full list containing 137 identified clusters is found in Supplementary Data 2, “DAVID PrF-PF”. ^1^enrichment score, geometric mean of member’s *P* values of the corresponding annotation cluster in -log10 scale of the annotation cluster; ^2^number of gene counts, ^3^ Fold-change, ^4^Benjamini-Hochberg value, and ^5^false discovery rate (*P* value adjusted). *internal gene ID defines a unique gene cluster belonging to a gene entry.

Functional annotation clustering of the 154 PF-SF DEGs resulted in 128 DAVID IDs in 27 clusters (Supplementary Data S2). Identified terms included “reproductive process”, “cellular response to chemical stimulus”, “ovulation cycle process”, and “epithelial cell proliferation” (Table 2). The KOs included terms involved in “focal adhesion” and “inositol phosphate metabolism” along with “sphingolipid”, “phosphatidylinositol”, “thyroid hormone”, “calcium”, “vascular endothelial growth factor (VEGF)”, “ErbB” and “hypoxia-inducible factor 1 (HIF-1)” signalling pathways (Table 2). Additionally, “β-catenin”, “apelin”, “extra-nuclear estrogen”, “IL8 CXCR1”, “RAS”, and “TXA2” signalling were identified. Lastly, “E2F”, “forkhead box O (FOXO)”, “Hedgehog”, “IL6 JAK STAT3”, “insulin-like growth factor (IGF)”, “KIT”, “Notch”, and “Wnt” were identified though not significantly enriched (Supplementary Data S3).

**Table 2:** Functional annotation clusters identified during the PF-SF transition in domestic cat using DAVID.

Cluster	ES^1^	Description (DAIVD ID*)	Count^2^	FC^3^	BH^4^	FDR^5^
1	1.76	Reproductive process	15	2.5	5.8E−1	3.1E0
2	1.73	Phospholipase activity	4	8.0	8.7E−1	1.6E1
3	1.7	Cellular response to chemical stimulus	20	1.7	9.3E−1	2.3E1
4	1.52	Ion binding	31	1.5	7.8E−1	1.8E1
5	1.38	Sphingolipid signalling pathway	5	5.2	9.3E−1	1.7E1
		Fc gamma R-mediated phagocytosis	4	6.1	8.0E−1	2.8E1
		Choline metabolism in cancer	3	3.7	6.6E−1	9.2E1
6	1.31	Fc gamma R-mediated phagocytosis	4	6.1	8.0E−1	2.8E1
		Leukocyte transendothelial migration	4	4.4	5.3E−1	5.3E1
		Focal adhesion	5	3.0	5.5E−1	6.3E1
7	1.27	Cell adhesion	17	2.5	8.6E−1	1.8E0
8	1.25	Sphingolipid signalling pathway	5	5.2	9.3E−1	1.7E1
		Inflammatory mediator regulation of TRP channels	4	5.5	5.9E−1	3.4E1
		Phosphatidylinositol signalling system	4	5.1	6.1E−1	4.0E1
		Cholinergic synapse	4	4.7	5.8E−1	4.8E1
		Thyroid hormone signalling pathway	4	4.5	5.4E−1	5.2E1
		Oxytocin signalling pathway	4	3.5	5.7E−1	7.3E1
		Inositol phosphate metabolism	3	5.3	5.8E−1	7.5E1
		Calcium signalling pathway	4	2.9	6.3E−1	8.8E1
9	1.25	Cytosolic transport	4	5.4	9.2E−1	4.8E1
10	1.24	Extracellular region	34	1.4	9.4E−1	2.9E1
11	1.23	Sphingolipid signalling pathway	5	5.2	9.3E−1	1.7E1
		VEGF signalling pathway	3	6.3	5.4E−1	6.3E1
		HIF-1 signalling pathway	3	3.9	6.5E−1	9.1E1
12	1.17	Ovulation cycle process	3	8.7	9.3E−1	5.5E1
13	1.15	Serine hydrolase activity	5	4.3	7.6E−1	3.1E1
14	1.14	Regulation of angiogenesis	5	4.3	9.3E−1	4.0E1
15	1.14	Epithelial cell proliferation	6	3.3	9.3E−1	4.6E1
16	1.12	ErbB signalling pathway	4	5.8	6.7E−1	3.1E1
		Focal adhesion	5	3.0	5.5E−1	6.3E1
		Proteoglycans in cancer	4	2.6	6.6E−1	9.3E1
17	1.09	Serotonergic synapse	4	4.9	5.7E−1	4.4E1
		Cholinergic synapse	4	4.7	5.8E−1	4.8E1
		Glutamatergic synapse	4	4.6	5.3E−1	4.9E1
		Circadian entrainment	3	4.0	6.4E−1	8.9E1
		Retrograde endocannabinoid signalling	3	3.9	6.5E−1	9.1E1
18	1.09	Somatodendritic compartment	7	3.2	9.9E−1	2.4E1
19	1.07	Phosphorus metabolic process	22	1.6	9.3E−1	4.3E1
20	1.06	Regulation of angiogenesis	5	4.3	9.3E−1	4.0E1

The top 20 categories grouped by similar GO and KO terms are listed. The full list containing 27 identified clusters is found in Supplementary Data 2, “DAVID PF-SF”.

*Internal gene ID defines unique gene cluster belonging to a gene entry.

^1^Enrichment score, geometric mean of member’s *P* values of the corresponding annotation cluster in − log10 scale of the annotation cluster.

^2^Number of gene counts.

^3^Fold-change.

^4^Benjamini-Hochberg value.

^5^False discovery rate (*P* value adjusted).

**Figure 3:**
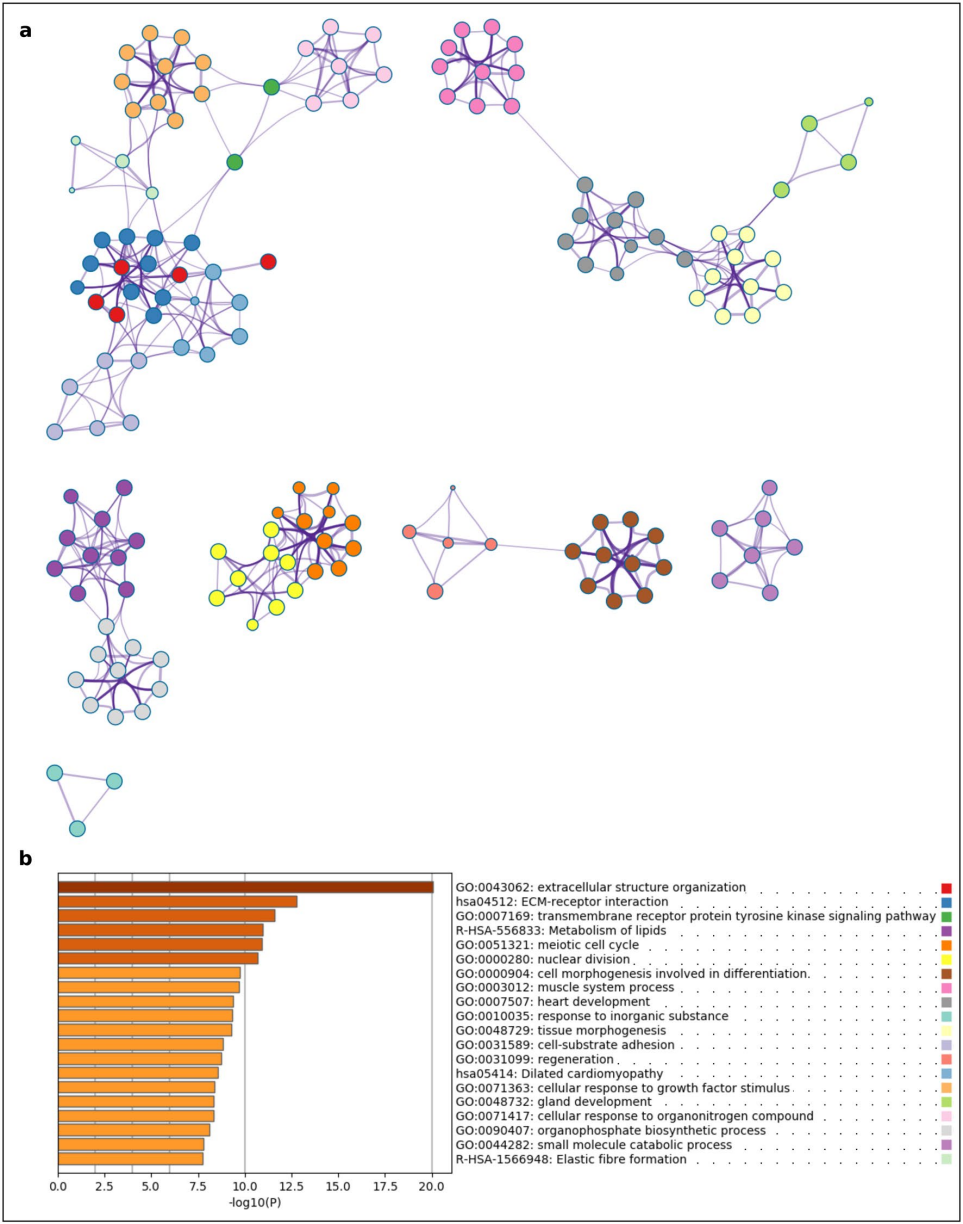
Functional enrichment analyses of differentially expressed genes (DEGs) during the primordial-to-primary ovarian follicle transition in domestic cat. **(a)** The enrichment ontology cluster graph represents each term as a circle, the size of the circle is proportional to the number of DEGs associated with that term, each cluster is coloured uniquely meaning that circles of the same colour are associated with the same cluster. The edges connect terms that have a similarity score of > 0.3 which influences the density of the edge line; **(b)** bar chart of GO and KO terms coloured by *P* values. Metascape (http://metascape.org) was utilised for visualisation.

**Figure 4:**
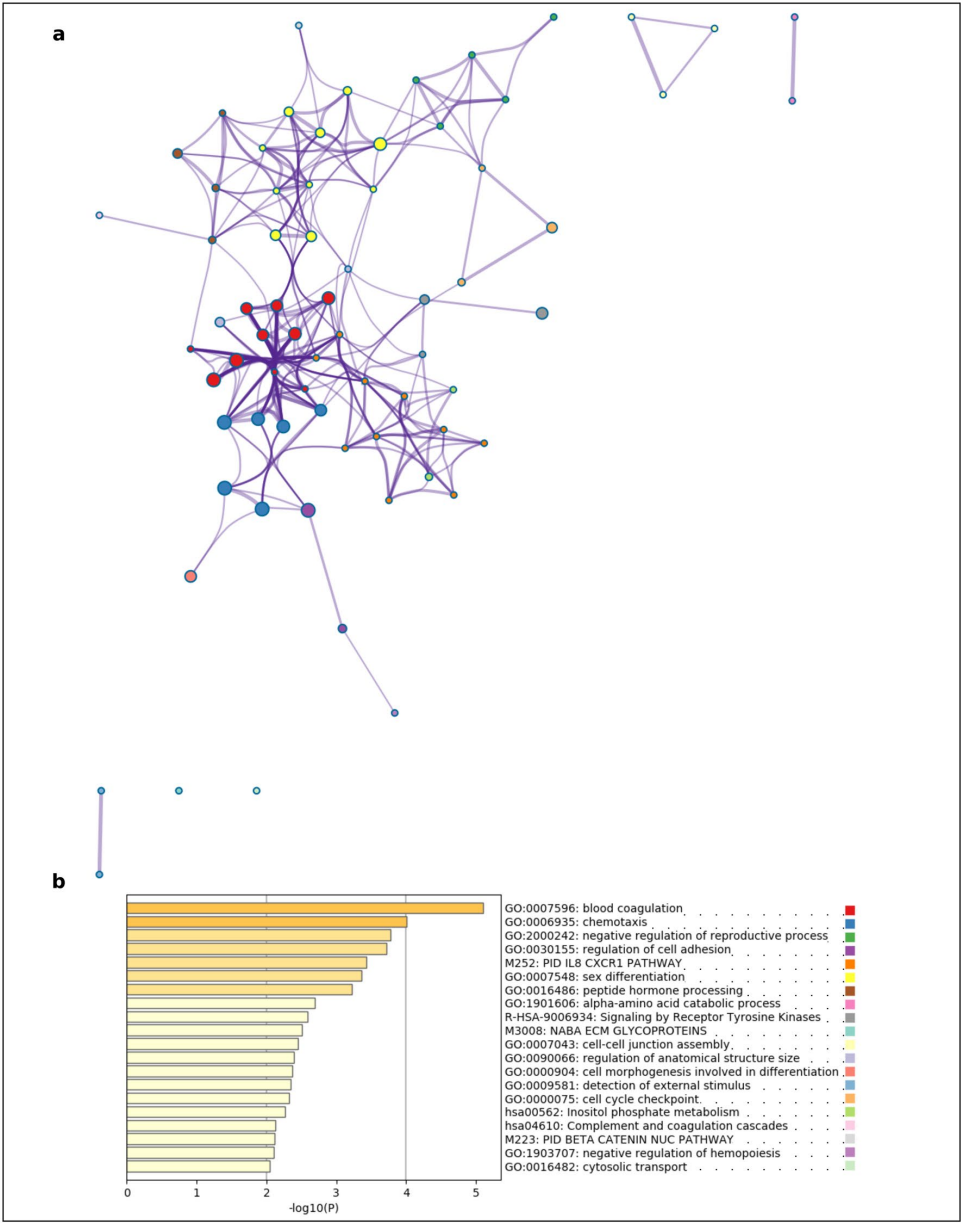
Functional enrichment analyses of differentially expressed genes (DEGs) during the primary-to-secondary ovarian follicle transition in domestic cat. **(a)** The enrichment ontology cluster graph represents each term as a circle, the size of the circle is proportional to the number of DEGs associated with that term, each cluster is coloured uniquely meaning that circles of the same colour are associated with the same cluster. The edges connect terms that have a similarity score of > 0.3 which influences the density of the edge line; **(b)** bar chart of GO and KO terms coloured by *P* values. Metascape (http://metascape.org) was utilised for visualisation.

As described in the methods section, Metascape generated functional annotation network clusters and bar plots of enriched ontology terms for visualisation. The results from Metascape were extracted for PrF-PF (Fig. 3 and Supplementary Fig. S5) and PF-SF (Fig. 4 and Supplementary Fig. S6), respectively. Based on Metascape analysis, the functional annotation cluster and enrichment PrF-PF and PF-SF results were summarised in Excel format, respectively (Supplementary Data S3 and S4, respectively). The following pathways, processes, and regulators were selected for further analysis: PI3K-Akt; TGF-β; erythroblastoma (ErbB); HIF-1 signalling pathways; over-represented biological processes associated with the extracellular matrix (ECM); and potential structural regulators of ovarian folliculogenesis such as, the dynein complex.

### Selected RNA-sequencing transcript expression

**Figure 5:**
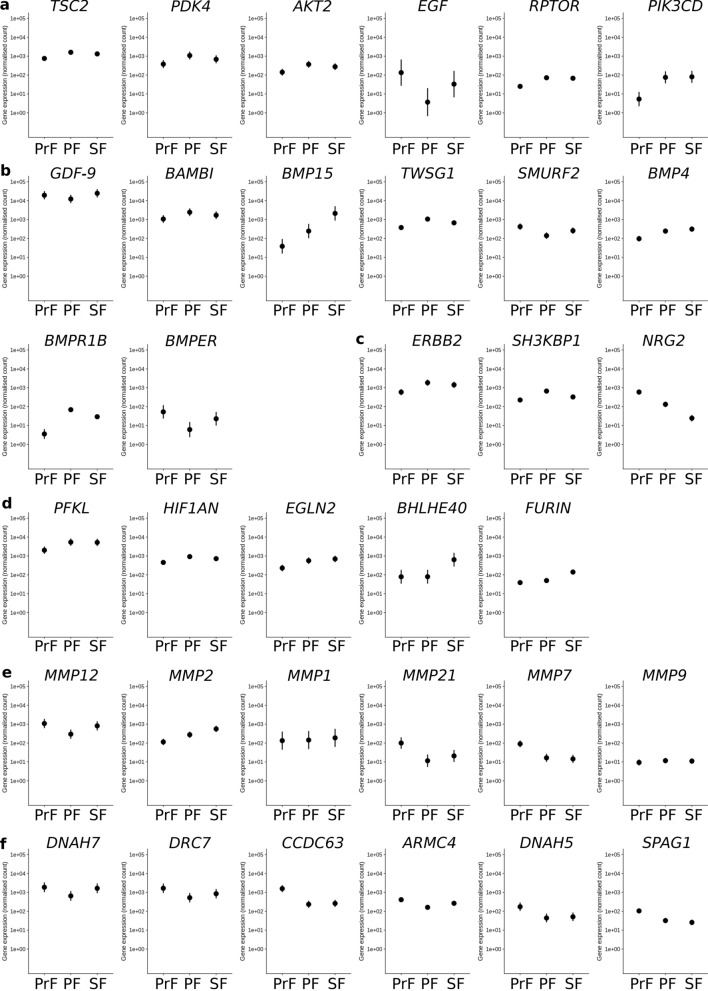
Transcript expression plots of normalised counts for genes of interest from preantral ovarian follicles in domestic cat (RNA-sequencing data). **(a)** Genes associated with the PI3K-Akt pathway from left-to-right: TSC complex subunit 2 (*TSC2*); pyruvate dehydrogenase kinase 4 (*PDK4*); AKT serine/threonine kinase 2 (*AKT2*); epidermal growth factor (*EGF*); regulatory associated protein of mTOR complex 1 (*RPTOR*); and PI3K subunit delta (*PIK3CD*); **(b)** TGF-β pathway from left-to-right: growth differentiation factor 9 (*GDF-9*); BMP and activin membrane bound inhibitor (*BAMBI*); bone morphogenetic protein 15 (*BMP15*); twisted gastrulation BMP signalling modulator 1 (*TWSG1*); SMAD specific E3 ubiquitin protein ligase 2 (*SMURF2*); *BMP4*; bone morphogenetic protein receptor type 1B (*BMPR1B*); and BMP binding endothelial regulator (*BMPER*); **(c)** erythroblastoma (ErbB) pathway: Erb-B2 receptor tyrosine kinase 2 (*ERBB2*); SH3 domain containing kinase binding protein 1 (*SH3KBP1*); and ErbB factor neuregulin 2 (*NRG2*); **(d)** hypoxia-inducible factor 1 (HIF-1) pathway: phosphofructokinase, liver type (*PFKL*); hypoxia inducible factor 1 subunit alpha inhibitor (*HIF1AN*); egl-9 family hypoxia inducible factor 2 (*EGLN2*); basic helix-loop-helix family member e40 (*BHLHE40*); and furin, paired basic amino acid cleaving enzyme (*FURIN*); **(e)** matrix metalloproteinase (MMP): *MMP12*; *MMP2*; *MMP1*; *MMP21*; *MMP7*; and *MMP9*; **(f)** axonemal dynein complex: dynein axonemal heavy chain 7 (*DNAH7*); dynein regulatory complex subunit 7 (*DRC7*); coiled-coil domain containing 63 (*CCDC63*); armadillo repeat containing 4 (*ARMC4*); dynein axonemal heavy chain 5 (*DNAH5*); and sperm associated antigen 1 (*SPAG1*) in PrF, PF, and SFs. Gene expression of negative binomial general linearised model normalised data.

Supplementary Data S3 and S4 identified which genes were associated with the aforementioned selection, respectively. Several genes were selected (investigating both the significant and non-significant differential expression gene lists), transcript expression levels were plotted, and DESeq2 results were extracted for each (Fig. 5 and Table 3). Some genes were selected due to functional implications related to ovarian folliculogenesis in other species. Whereas, others such as, bone morphogenetic proteins (BMPs) and MMPs were selected due to the piling evidence describing the essential role of these factors during mammalian ovarian folliculogenesis. For each numbered section below the following genes are listed in order of decreasing “baseMean” values: (1) PI3K-Akt signalling pathway: TSC complex subunit 2 (*TSC2*); pyruvate dehydrogenase kinase 4 (*PDK4*); AKT serine/threonine kinase 2 (*AKT2*); epidermal growth factor (*EGF*); regulatory associated protein of mTOR complex 1 (*RPTOR*); and PI3K subunit delta (*PIK3CD*) (Fig. 5a). Transcript expression increased for each of these genes except *EGF* during the PrF-PF transition (Table 3, Fig. 5a). In contrast, the non-significantly differentially expressed gene *EGF* decreased sharply during the PrF-PF transition only to increase sharply during the PF-SF transition (Fig. 5a). (2) TGF-β signalling pathway: growth differentiation factor 9 (*GDF-9*); BMP and activin membrane bound inhibitor (*BAMBI*); *BMP15*; twisted gastrulation BMP signalling modulator 1 (*TWSG1*); SMAD specific E3 ubiquitin protein ligase 2 (*SMURF2*); *BMP4*; bone morphogenetic protein receptor type 1B (*BMPR1B*); and BMP binding endothelial regulator (*BMPER*) (Table 3, Fig. 5b). Overall, the genes associated with the TGF-β signalling pathway demonstrated the most dynamic transcript expression. The highest “baseMean” value was estimated for *GDF-9* however, this gene was not significantly differentially expressed (Table 3). Transcript expression of *GDF-9* was in a comparable range throughout each ovarian follicle stage (Fig. 5b). *BAMBI, BMP15, TWSG1*, *BMP4* and *BMPR1B* showed a significant increase from PrF to PF (Fig. 5b). There was an oppositional pattern observed for *SMURF2* and *BMPER* (Fig. 5b). Transcript counts of *BMP4* and *BMP15* increases towards SF too but this was not significant (Fig. 5b). (3) ErbB signalling pathway: Erb-B2 receptor tyrosine kinase 2 (*ERBB2*); SH3 domain containing kinase binding protein 1 (*SH3KBP1*); and ErbB factor neuregulin 2 (*NRG2*) (Fig. 5c). A sequential decrease all the way until the SF stage was observed for *NRG2*. In contrast, expression of *ERBB2* and *SH3KBP1* transcripts increases from PrF to PF stage (Fig. 5c, Table 3). (4) HIF-1 signalling pathway: phosphofructokinase, liver type (*PFKL*); hypoxia inducible factor 1 subunit alpha inhibitor (*HIF1AN*); egl-9 family hypoxia inducible factor 2 (*EGLN2*); basic helix-loop-helix family member e40 (*BHLHE40*); and furin, paired basic amino acid cleaving enzyme (*FURIN*) (Fig. 5d). Transcript counts for three of them (*PFKL*, *HIFNAN*, *EGLN2*) increased significantly during PrF-PF transition whereas the other two increased strongly in the PF-SF transition (Fig. 5d, Table 3). (5) Variable transcript expression patterns were found in the MMP group: *MMP12*, *MMP21* and *MMP7* expression decreased in PrF-PF then increased in PF-SF; *MMP2* demonstrated an increase in expression in this transition; *MMP1* had a comparable transcript expression throughout each stage and was not significantly differentially expressed; and *MMP9* showed a low but unchanged expression level too (Fig. 5e). (6) The dynein genes included: dynein axonemal heavy chain 7 (*DNAH7*); dynein regulatory complex subunit 7 (*DRC7*); coiled-coil domain containing 63 (*CCDC63*); armadillo repeat containing 4 (*ARMC4*); dynein axonemal heavy chain 5 (*DNAH5*); and sperm associated antigen 1 (*SPAG1*) (Fig. 5f). All counts were highest in PrFs in comparison to PFs. Values between PF and SF were comparable (Table 3).

**Table 3:** Transcript expression DESeq2 data from preantral ovarian follicles in domestic cat (RNA-sequencing data).

Gene name *symbol*	baseMean	PrF-PF		PF-SF	
		FC	padj	FC	padj
**PI3K-Akt pathway**					
TSC complex subunit 2 TSC2*	1221.48	− 1.06	0.02	0.28	0.93
Pyruvate dehydrogenase kinase 4 PDK4*	711.73	− 1.53	0.02	0.66	0.77
AKT serine/threonine kinase 2 AKT2*	262.00	− 1.34	0.04	0.41	0.87
Epidermal growth factor *EGF*	56.92	5.21	0.12	− 3.18	0.75
Regulatory associated protein of mTOR complex 1 RPTOR*	54.76	− 1.4	0.03	0.08	1.00
PI3K subunit delta PIK3CD*	53.64	− 3.31	0.00	− 0.10	0.99
**TGF-β pathway**					
Growth differentiation factor 9 *GDF-9*	18,699.46	0.65	0.33	− 1.00	0.48
BMP and activin membrane bound inhibitor BAMBI*	1734.87	− 1.22	0.05	0.53	0.81
Bone morphogenetic protein 15 BMP15*	801.53	− 2.83	0.05	− 3.11	0.15
Twisted gastrulation BMP signalling modulator 1 TWSG1*	701.81	− 1.47	0.00	0.66	0.58
SMAD specific E3 ubiquitin protein ligase 2 SMURF2*	273.55	1.6	0.01	− 0.88	0.50
Bone morphogenetic protein 4 BMP4*	218.04	− 1.2	0.04	− 0.35	0.88
Bone morphogenetic protein receptor type 1B BMPR1B*	33.97	− 3.83	0.00	1.23	0.72
BMP binding endothelial regulator BMPER*	27.34	3.03	0.03	− 1.91	0.55
**ErbB pathway**					
Erb-B2 receptor tyrosine kinase 2 ERBB2*	1288.61	− 1.6	0.00	0.38	0.87
SH3 domain containing kinase binding protein 1 SH3KBP1*	406.38	− 1.54	0.00	1.03	0.15
ErbB factor neuregulin 2 NRG2*	250.12	2.12	0.00	2.36	0.00
**HIF-1 pathway**					
Phosphofructokinase, liver type PFKL*	4193.36	− 1.4	0.01	0.05	1.00
Hypoxia inducible factor 1 subunit alpha inhibitor HIF1AN*	698.78	− 1.04	0.00	0.35	0.76
Egl-9 family hypoxia inducible factor 2 EGLN2*	496.63	− 1.22	0.04	− 0.30	0.93
Basic helix-loop-helix family member e40 BHLHE40*	265.23	0.01	0.90	− 2.99	0.03
Furin, paired basic amino acid cleaving enzyme FURIN*	76.47	− 0.3	0.83	− 1.49	0.04
**Extracellular matrix**					
Matrix metalloproteinase 12 MMP12*	729.45	1.88	0.01	− 1.46	0.23
Matrix metalloproteinase 2 MMP2*	316.92	− 1.28	0.04	− 1.02	0.35
Matrix metalloproteinase 1 *MMP1*	155.42	− 0.07	1	− 0.38	0.97
Matrix metalloproteinase 21 MMP21*	44.25	3.06	0.01	− 0.85	0.90
Matrix metalloproteinase 7 MMP7*	40.47	2.51	0.00	0.17	1.00
Matrix metalloproteinase 9 *MMP9*	10.73	− 0.11	1	0.05	1
**Axonemal dynein complex**					
Dynein axonemal heavy chain 7 DNAH7*	1381.74	1.51	0.04	− 1.34	0.33
Dynein regulatory complex subunit 7 DRC7*	1006.22	1.67	0.02	− 0.70	0.78
Coiled-coil domain containing 63 CCDC63*	689.57	2.74	0.00	− 0.15	0.98
Armadillo repeat containing 4 ARMC4*	277.97	1.35	0.00	− 0.71	0.48
Dynein axonemal heavy chain 5 DNAH5*	89.48	2.01	0.02	− 0.24	0.99
Sperm associated antigen 1 SPAG1*	54.02	1.66	0.02	0.37	0.94

The transcript expression data was extracted from DESeq2 analysis and were summarised in order of decreasing “baseMean” values. “PI3K-Akt pathway” genes: *TSC2*; *PDK4*; *AKT2*; *EGF*; *RPTOR*; and *PIK3CD*. “TGF-β pathway” genes: *GDF-9*; *BAMBI*; *BMP15*; *TWSG1*; *SMURF2*; *BMP4*; *BMPR1B*; *BMPER*. “ErbB pathway” genes: *ERBB2*, *SH3KBP1*, and *NRG2*. “HIF-1 pathway” genes: *PFKL*; *HIF1AN*; *EGLN2*; *BHLHE40*; *FURIN*. “Extracellular matrix” genes: *MMP12*; *MMP2*; *MMP1*; *MMP21*; *MMP7*; and *MMP9.* “Axonemal dynein complex” genes: *DNAH7*; *DRC7*; *CCDC63*; *ARMC4*; *DNAH5*; and *SPAG1*. “baseMean” is the average of the normalised count values, dividing by size factors, taken over all samples; “FC” fold-change is log2FoldChange, the effect size estimate. This value indicates how much the gene or transcript's expression seems to have changed between the contrasts reported on a logarithmic scale to base 2; “padj” is the adjusted *P* value for multiple testing for the gene or transcript; PrF-PF and PF-SF denote primordial versus primary and primary versus secondary DESeq2 contrasts; and * indicates differential gene expression significance with an adjusted *P* value of < 0.05 and log2 fold-change ≥ 1.

### qRT-PCR comparative analysis

The genes, *BMP15* and histone H1t (*HIST1H1T)*, were compared to the RNA-sequencing data by evaluating relative gene expression trends with qRT-PCR data. Diagnostic plots with RNA-sequencing data of selected genes demonstrated an increase in *BMP15* gene expression as ovarian follicles developed from PrFs toward SFs (Supplementary Fig. S7a). A decrease in *HISTH1T* gene expression was observed as ovarian follicles progressed from the PrF stage onward (Supplementary Fig. S7a). The negative binomial general linearised model of normalised RNA-sequencing data showed a similar increase in *BMP15* gene expression as the ovarian follicles developed (Supplementary Fig. S7b, above). Applying the same approach to *HISTH1T*, gene expression was detected in PrF only (Supplementary Fig. S7b, below). Analysis of *BMP15* by qRT-PCR revealed that gene expression was nearly undetectable in PrFs and PFs however, *BMP15* gene expression was observed in SFs (Supplementary Fig. S7b, above right). The Kruskal–Wallis test detected significant changes of gene expression throughout ovarian follicular development (*P* value = 0.04627), but the subsequent post-hoc test reported only a tendency for higher expression in SF compared to PrF and PF. In contrast, a high relative gene expression of *HIST1H1T* was found in PrFs which decreased considerably as ovarian follicles progressed toward PF and SF stages, the differences of PrF and PF as well as PrF and SF were confirmed as statistically significant (Supplementary Fig. S7b, below right).

## Discussion

As far as we are aware, this is the first whole ovarian follicle transcriptomic analysis during primordial ovarian follicle (PrF) development and early follicular growth during the primary (PF) to secondary (SF) ovarian follicle transition in a non-rodent animal. In contrast to most gene expression studies performed on adult ovaries, in this study the ovarian follicle is considered as a functional unit. There was no differentiation made between oocytes and the surrounding ovarian follicular cells. This is primarily due to the fact that after isolation from the ovarian cortex, dissection of oocytes from early ovarian follicles is impossible without any severe damage. However, laser dissection microscopy (LCM) is an alternative method which may circumvent such problems and has thus far, been executed in human^20,49^ and sheep^16,17^. Nevertheless, the use of whole ovarian follicles as a source for transcriptome analysis is essential to monitor follicular growth in vitro*.*

The initial RNA-sequencing analysis revealed a higher proportion of differentially expressed genes (DEGs) during the activation of dormant ovarian follicles (PrF-PF: 2,226 DEGs) in comparison to the growth phase (PF-SF: 156 DEGs) (Fig. 2). Analysing transcripts of early stages of human oocytes, 223 and 268 genes were detected as significantly expressed in oocytes from PrFs and PFs, respectively^50^. The differences in differential gene expression levels may be due to the use of whole ovarian follicles in comparison to the latter oocyte-specific approach. Another group analysed oocytes and granulosa cells (GCs) of ovine ovarian follicles separately^17^. In this study, less than 200 DEGs in oocytes and GCs during the PrF-PF transition were detected. In contrast to this data, a higher number of gene expression changes during the PF-SF transition were observed—around 500 DEGs in oocytes and 400 DEGs in GCs^17^. So far it is not clear if the discrepancies in the number of DEGs are species-specific or due to the particular analytical approach such as, intact ovarian follicles versus laser dissected ovarian follicles from tissue slices. Additionally, we must consider that in this study the ratio of expressed genes of oocyte origin is decreasing with ovarian follicular growth. An intact PrF contains < 20 follicular cells per oocyte, whereas, this ratio is shifted to 1:50 for PF and 1:200 SFs, respectively^51^. To account for the number of ovarian follicular cells the number of follicles per pool were adapted (PrF n = 180, PF n = 45, and SF n = 9). Therefore, differential gene expression in PF-SF is mirroring more GC expression than oocyte expression, maybe leading to a lower number of detectable DEGs than in PrF-PF. In the future, it will be worthwhile to consider single cell transcriptomics to investigate gene expression levels individually.

After differential gene expression analysis the gene lists were functionally annotated resulting in the identification of over-represented GO and KO terms. As described previously, conserved signalling pathways and biological processes (BPs) that were over-represented were considered. This included the two signalling pathways predominantly studied during ovarian folliculogenesis in other mammals: the PI3K-Akt and TGF-β pathways. Additionally, signalling pathways more ambiguously described though implicated in ovarian folliculogenesis such as, ErbB and HIF-1 pathways were given attention too. The PI3K-Akt signalling pathway within the oocyte is a key regulator of PrF activation and has been described during ovarian folliculogenesis in bovine, human, ovine, and porcine^52^. Currently, it is one of the major non-gonadotrophic growth factor pathways regulating ovarian follicles. As expected, the PI3K-Akt signalling pathway was identified during the PrF-PF transition in domestic cat (Table 2). The TGF-β signalling pathway was identified in the PrF-PF data under the parent GO term “transmembrane receptor protein serine/threonine kinase signalling pathway” (Table 2). Similarly to the PI3K-Akt pathway, the TGF-β signalling pathway has been studied during ovarian folliculogenesis in bovine, human, ovine, porcine, rodents, and rhesus monkeys^53^. For the domestic cat, the development of ovarian follicles in vitro with the supplementation of PI3K-Akt and TGF-β-associated factors has been investigated^9,54^. The studies focused on the effect of epidermal growth factor (EGF), its receptor (EGFR), and the growth differentiation 9 factor (GDF-9) supplementation either in combination or alone. Signalling via EGF and EGFR up-regulates the PI3K pathway^55^. In goat, EGF stimulates in vitro growth during the PrF-PF transition^56^ and in rat, it is implicated in the growth of PrFs toward the SF stage^57^. The TGF-β factor, GDF-9, has been shown to have a similar influence on preantral ovarian follicles in several mammals. In vitro studies have implicated GDF-9 supplementation in PrF activation and ovarian follicle viability in human^58^, goat^59,60^, bovine^60^, and hamster^61^. Additionally, GDF-9 has an over-arching function within the PI3K pathway in rat preantral ovarian follicles^62^. Interestingly, for the domestic cat, the culture of ovarian cortical slices in medium supplemented with EGF and/or GDF-9 have shown that EGF but not GDF-9 improved follicle viability^54^. Medium containing GDF-9 not only had no beneficial influence on ovarian tissues but negligibly impacted ovarian follicle viability^54^. Interestingly, although the transcriptomic data for *GDF-9* was determined with a high “baseMean” value it was not significantly differentially expressed (Table 3). Therefore, for the domestic cat it is likely to be more essential in later stages instead. This is the case in the rat where GDF-9 supplementation in vitro promotes ovarian follicular growth only after the PF stage^63,64^. Other functionally annotated factors have also been studied in other models during ovarian folliculogenesis. This includes BMP4 which is also described in rats to function as an ovarian follicle survival factor promoting PrF development^65^ and in humans, where BMP4 is implicated in regulating ovulation via remodelling of the ovarian extracellular matrix (ECM) by facilitating in cumulus-oocyte-complex and mural GC separation^66^. A comparative analysis was performed on *BMP15* with the qRT-PCR method. The RNA-sequencing transcript counts of *BMP15* were shown to increase from the PF stage onward (Supplementary Fig. S7). Similarly, BMP15 mRNA increased in expression with ongoing follicular development (Supplementary Fig. S7). This supports previous studies in other species such as, human and rat where increased BMP15 expression promotes GC proliferation and theca layer development^67^.

As mentioned, the ErbB and HIF-1 signalling pathways were included as mechanisms of interest. Currently, there are no studies into the role of these pathways during ovarian folliculogenesis in the domestic cat. However, there is evidence in other species of its involvement in ovarian follicle development. Briefly, the ErbB has recently been found to be down-regulated in human oocytes during the PrF-PF transition^20^. Previously, the ErbB factor neuregulin 1 (NRG1) has been investigated in mural GCs and theca cells from rodent periovulatory follicles^68–70^. In the domestic cat, *NRG2* transcript expression was observed along with other ErbB-associated factors such as, *ERBB2* and *SH3KBP1* (Table 3). In contrast to the rodent model, not only was a different variant found in the domestic cat but a sequential decrease of *NRG2* expression was observed all the way until the SF stage (Fig. 5c). Investigations into the HIF-1 pathway may also provide an alternative insight into preantral ovarian folliculogenesis in the domestic cat. Some factors identified had either similar or higher “baseMean” values than those identified for the TGF-β pathway (Table 3). For example, *PFKL* had the highest “baseMean” overall the factors (excluding *GDF-9*) (Table 3). Interestingly, each HIF-1 signalling factor demonstrated a tendency to increase in transcript expression during ovarian follicle development (Fig. 5d). In other species, increased HIF-1α expression in GCs is implicated in regulating follicular growth and development in rats^71^. Additionally, HIF-1 signalling in bovine primary GCs is suggested to be involved in steroidogenesis and cell proliferation during follicular development^72^. Although not extensively described here it will be interesting to study these pathways in relation to domestic cat ovarian folliculgenesis in the future.

Noteworthy, “ECM-receptor interaction” and “focal adhesion” were identified within the same functional annotation cluster as “PI3K-Akt signalling pathway” (Table 1). During the development of ovarian follicles the ECM undergoes significant compositional remodelling. Upon the initial activation of PrFs, GCs become cuboidal, forming a PF, and the ovarian follicle moves toward the medullar region and away from the cortical region of the ovary^73^. Previously, in vivo imaging windows in mouse ovaries have revealed that collagen fibers can function as migration tracks for infiltrating tumor cells^74^. Additionally, 3D imaging analyses have revealed that the inward movement of ovarian follicles is likely due to the stiffer cortical enclosure as compared with the softer inner medulla^75^. Furthermore, this analysis demonstrated that ovarian follicles are in close contact to each other^75^. Thus, the migration of activated PrFs and developing preantral ovarian follicles may occur through a collective collaboration based on collagen fiber tracking, morphological reactions, and interactions between neighbouring ovarian follicles. Regarding the ECM, degradation occurs via matrix MMPs. This allows for continued enlargement of the developing ovarian follicle. In the ovary, MMP2 and MMP9 expression has been observed in rat, sheep, mouse, rhesus macaque, horse, cow, and human; MMP1 and MMP13 in rabbit, rat, horse, and rhesus macaque; and MMP7 mRNA in macaque pre-ovulatory GCs^76^. In the domestic cat, MMP1, MMP2, MMP3, MMP7, MMP9, and MMP13 mRNA are detectable at every developmental stage, and the abundance and expression of these enzymes was consistently dynamic^76^. The domestic cat transcriptomic data also demonstrated highly dynamic expression patterns throughout each MMP (*MMP2*, *MMP7*, *MMP12*, and *MMP21*) with *MMP2* showing a clear tendency to increase whereas, *MMP7*, *MMP12*, and *MMP21* decrease during ovarian folliculogenesis (Fig. 5e). Additionally, two MMP transcripts, *MMP12* and *MMP21*, not previously described for the domestic cat were identified. A higher level of transcript expression was observed for *MMP12* than *MMP21* (Fig. 5e). Additionally, the highest “baseMean” value overall was observed for *MMP12* (Table 3) therefore, it may be interesting to perform downstream mRNA analysis on this factor in the domestic cat. Subsequently, the changes that occur within the ECM subsequently affect nuclear dynamics through changes in lamina composition, membrane tension, and nuclear pore size which regulates chromatin configuration and gene expression^77^. Currently, these changes are not well understood for all species in respect to ovarian folliculogenesis. Screening for factors involved in oocyte dormancy with RNA-sequencing revealed that microenvironmental compression elicited a nuclear rotation response which was mediated by a motor protein called dynein in mice^73^. The inhibition of dynein significantly increased the number of growing follicles demonstrating its essential role in follicle dormancy in cultured murine ovaries^73^. Differentially expressed genes of the axonemal dynein complex were identified in the PrF-PF transition only (Supplementary Data S2). The heavy chain regions of the dynein complex contain the motor domain which is capable of producing movement in vitro^78^. In the transcriptomic data, the *DNAH5* and *DNAH7* heavy chains transcripts were identified along with the regulatory *DRC7* and structural factors *CCDC63* and *ARMC4* (Fig. 5f and Table 3). Thus, the two heavy chain transcripts, *DNAH5* and *DNAH7*, from the nine major phylogenetically classed dynein heavy chains may be involved in PrF dormancy and/or activation in the domestic cat. This, of course, needs further investigation in the future. Additionally, very high expression of histone *HIST1H1T* (H1.6) in PrFs was observed (Supplementary Fig. S7). H1 histones belong to the so-called linker histones, meaning that they are not part of the histone-octomer-centre of nucleosomes but connect to the ends of DNA that coils around such a centre leading to chromatin compaction. Furthermore, H1 also functions through interactions with other proteins that will in turn modify chromatin or take part in DNA-based processes like transcription^79^. Besides versions of H1 (H1.1 to H1.5) which are present in most somatic cells, other subtypes exist^79^, some of which are germ-line variants^80^. HIST1H1T was so far described as a testis-specific variant^81^, but could be detected in tumour cell lines, mouse embryonic stem cells and some normal somatic cells also^82^. To our knowledge, this is the first description of its presence in ovarian follicles, meaning that it could be present in female germ cells. Male H1 germ variants like H1T appear after spermatogonia stop proliferating and before transition proteins are detected and histones are replaced by protamines at late spermatid stages. H1T is the first variant to be expressed in meiotic spermatocytes, the presence in later germ cell stage up to elongated spermatids seems to be species-specific^80^. The dormant PrFs are arrested in meiosis I^83^ and primary spermatocytes are in prophase of meiosis I^84^. It could be possible that H1T fulfils a specific function during this cell division phase, for example, on chromatin structure. It is discussed that H1T supports the highly decondensed chromatin stage in early spermatocyte^80^. However, is has been shown that H1T repressed at least ribosomal DNA transcription by condensing chromatin structure^82^.

## Conclusion

RNA-sequencing analysis of ovarian follicles in domestic cat contributed to the increasing knowledge on factors and processes which regulate the recruitment and growth of ovarian follicles. Many biological processes were comparable to known data obtained for species such as; human however, species-specific features for the domestic cat may be present. In this study, the analysis focused on signalling factors and pathways along with mechanistic cues associated with the ECM and nuclear dynamics. Overall, the results are relevant to fundamental ovarian follicle developmental biology with an outlook toward developing techniques such as, IVG of ovarian follicles in the future.

## Supplementary Information

Supplementary Data S1.

Supplementary Data S2.

Supplementary Data S3.

Supplementary Data S4.

Supplementary Figure S1.

Supplementary Figure S2.

Supplementary Figure S3.

Supplementary Figure S4.

Supplementary Figure S5.

Supplementary Figure S6.

Supplementary Figure S7.

Supplementary Information 1.
