# Author Correction: Signalling pathways and mechanistic cues highlighted by transcriptomic analysis of primordial, primary, and secondary ovarian follicles in domestic cat

**DOI:** 10.1038/s41598-021-92241-9

**Published:** 2021-07-06

**Authors:** Shauna Kehoe, Katarina Jewgenow, Paul R. Johnston, Susan Mbedi, Beate C. Braun

**Affiliations:** 1 Reproduction Biology Department, Leibniz Institute for Zoo and Wildlife Research, Alfred‑Kowalke‑Straße 17, 10315 Berlin, Germany; 2 Berlin Center for Genomics in Biodiversity Research BeGenDiv, Königin‑Luise‑Straße 6‑8, D‑14195 Berlin, Germany; 3 Leibniz-Institute of Freshwater Ecology and Inland Fisheries, Müggelseedamm 310, 12587 Berlin, Germany; 4 Freie Universität Berlin, Institut Für Biologie, Königin‑Luise‑Straße 1‑3, 14195 Berlin, Germany; 5 Museum für Naturkunde, Invalidenstraße 43, 10115 Berlin, Germany

Correction to: *Scientific Reports* https://doi.org/10.1038/s41598-021-82051-4, published online 29 January 2021

The original version of this Article contained errors in the Acknowledgements section.

“We thank Anke Schmidt (IZW), Dr. Daniel Förster (IZW), and Sarah Sparmann (BeGenDiv) for technical assistance. The publication of this article was funded by the Open Access Fund of the Leibniz Association.”

now reads:

“We thank Anke Schmidt (IZW), Dr. Daniel Förster (IZW), and Sarah Sparmann (BeGenDiv) for technical assistance.”

The original Article has been corrected.

