# Supplementary material for: Signalling pathways and mechanistic cues highlighted by transcriptomic analysis of primordial, primary, and secondary ovarian follicles in domestic cat
Source: Sci Rep. 2021 Jan 29;11:2683. doi: 10.1038/s41598-021-82051-4 (PMC7846758; doi:10.1038/s41598-021-82051-4)
Supplement: Supplementary file 12 — Supplementary Information 1. [file 41598_2021_82051_MOESM12_ESM.docx]

**Signalling pathways and mechanistic cues highlighted by transcriptomic analysis of primordial, primary, and secondary ovarian follicles in domestic cat**

**Kehoe Shauna^1*^, Jewgenow Katarina^1^, Johnston Paul R^2,3^, Mbedi Susan^2,4^, Braun Beate C^1^**

^1^Leibniz Institute for Zoo and Wildlife Research, Reproduction Biology, Berlin, 10315, Germany

^2^Berlin Center for Genomics in Biodiversity Research, Berlin, D-14195, Germany

^3^Leibniz-Institute of Freshwater Ecology and Inland Fisheries, Berlin, 12587, Germany

^4^Museum für Naturkunde, Invalidenstraße 43, Berlin, 10115, Germany

[^*^kehoe@izw-berlin.de](mailto:*kehoe@izw-berlin.de)

**Supplementary Data 1 Differential gene expression analysis results from primordial versus primary (PrF-PF) and primary versus secondary (PF-SF) ovarian follicle type contrasts in the domestic cat.** Differential gene expression was estimated between the ovarian follicle contrasts PrF-PF and PF-SF, respectively. Differential expression was considered as significant with an adjusted *P* value (padj) of < 0.05 and log2 fold change ≥ 1. “baseMean” is the average of the normalised count values, dividing by size factors, taken over all samples; “log2FoldChange” is the fold change, the effect size estimate reported on a logarithmic scale to base 2. “lfcSE” is the standard error of the log2FoldChange estimate; “stat” is the Wald statistic; “pvalue” is the Wald test *P* value; “padj” is the Benjamini-Hochberg adjusted *P* value for multiple testing for the gene or transcript; and “weight” is the weight of the estimated *P* value as calculated by the Independent Hypothesis Weighting method. PrF-PF_DEG and PF-SF_DEG denotes differentially expressed genes per ovarian follicle type contrast, respectively.

**Supplementary Data 2 Functional terms and pathways identified within the differentially expressed gene (DEG) lists of the primordial versus primary (PrF-PF) and primary versus secondary (PF-SF) ovarian follicle contrasts in the domestic cat.** The functional annotation tools “Functional Annotation” and “Gene Functional Classification” from the web-based platform “The Database for Annotation, Visualization and Integrated Discovery” (DAVID) v6.8 (<https://david.ncifcrf.gov/>) determined gene annotation enrichment and functional annotation clustering from DEGs identified within the PrF-PF and PF-SF contrasts. The gene ontology (GO) terms biological process (BP), cellular component (CC), and molecular function (MF) along with KEGG (Kyoto Encyclopedia of Genes and Genomes) orthology (KO) terms were categorised into numbered functional annotation clusters. The functional annotation tool (FAT) filtered out broad GO terms based on measured specificity. “Enrichment Score” is the calculated geometric mean of EASE scores (a modified Fisher Exact *P* value for each enriched annotation terms) estimated for all enriched annotation terms; “Count” is the number of genes associated with a given term; “P_Value” is the *P* value from the modified Fisher’s exact test; “Fold Change” is the fold change; “Benjamini” is the adjusted *P* value of the Benjamini and Hochberg method; “FDR” is the adjusted *P* value controlled by the false discovery rate; “Input” describes the options selected prior to analysis.

**Supplementary Data 3 Gene annotation and functional enrichment of the differentially expressed genes (DEGs) identified within the primordial versus primary (PrF-PF) ovarian follicle contrast in the domestic cat.** Metascape analysis (<http://metascape.org>) generated two Excel spreadsheets containing gene annotation and functional enrichment results for the PrF-PF ovarian follicle DEG list input.

**Supplementary Data 4 Gene annotation and functional enrichment of the differentially expressed genes (DEGs) identified within the primary versus secondary (PF-SF) ovarian follicle contrast in the domestic cat.** Metascape analysis (<http://metascape.org>) generated two Excel spreadsheets containing gene annotation and functional enrichment results for the PF-SF ovarian follicle DEG list input.

**Supplementary Figure S1 Quality control statistics and log count normalisation box plots of domestic cat preantral ovarian follicles (RNA-sequencing data). (a)** The table shows the total sequence distribution between samples, the percentage of mapped reads (% aligned), and number of mapped reads in millions. Sample IDs denote primordial (PrF) (Sample_1-3_S1-3), primary (PF) (Sample_4-6_S4-6), and secondary ovarian follicle samples (SF) (Sample_7-9_S7-9); **(b)** log counts not normalised (above) and log counts normalised (plus a pseudocount) (below) were visualised.

**Supplementary Figure S2** **Principal component analysis demonstrates sample-to-sample correlation based on domestic cat ovarian follicle type.** The nine samples shown in the 2D plane demonstrates **(a)** the first two principal components with PC1 at 37% variance and PC2 at 16% variance for all genes; **(b)** the first and third principal component with PC1 at 37% variance and PC3 at 12% variance for all genes; and **(c)** the first two principal components with PC1 at 39% variance and PC2 at 19% variance for 500 genes. Ovarian follicle types are grouped as (red) primordial (PrF), (green) primary (PF), and (blue) secondary ovarian follicles (SF).

**Supplementary Figure S3** **Hierarchical cluster heatmap based on Euclidean distances demonstrates sample-to-sample relationships in preantral ovarian follicles from domestic cat.** **(a)** “A” denotes primordial (PrF), “B” primary (PF), and “C” secondary ovarian follicles (SF); **(b)** samples S1-3 consists of the PrF samples, S4-6 of PFs, and S7-9 of SFs. The colour scale indicates the degree of correlation.

**Supplementary Figure S4 Hierarchical cluster dendograms based on Euclidean distance matrix demonstrates sample-to-sample relationships in preantral ovarian follicles from domestic cat.** Sample_1-3_S1-3 primordial follicles; Sample_4-6_S4-6 primary follicles; and Sample_7-9_S7-9 secondary ovarian follicles.

**Supplementary Figure S5 Functional enrichment analysis of differentially expressed genes (DEGs) during the primordial-to-primary ovarian follicle transition in domestic cat defined by *P values.*** The enrichment ontology cluster graph represents each term as a circle, the size of the circle is proportional to the number of DEGs associated with that term; clusters are defined by *P values* from *Felis catus* Entrez IDs converted into *Homo sapiens* Ensembl IDs. The edges connect terms that have a similarity score of > 0.3 which influences the density of the edge line. Metascape (http://metascape.org) was used for visualisation.

**Supplementary Figure S6 Functional enrichment analysis of differentially expressed genes (DEGs) during the primary-to-secondary ovarian follicle transition in domestic cat defined by *P values.*** The enrichment ontology cluster graph represents each term as a circle, the size of the circle is proportional to the number of DEGs associated with that term; clusters are defined by *P values* from *Felis catus* Entrez IDs converted into *Homo sapiens* Ensembl IDs. The edges connect terms that have a similarity score of > 0.3 which influences the density of the edge line. Metascape (http://metascape.org) was used for visualisation.

**Supplementary Figure S7 Comparative analysis of RNA-sequencing transcript and qRT-PCR mRNA expression patterns in preantral ovarian follicles from domestic cat. (a)** Transcript expression plots of normalised counts for *BMP15* and *HIST1H1T* genes (RNA-sequencing data). The normalised counts plus a pseudocount of 0.5 were estimated; **(b)** negative binomial general linearised model of normalised data (left) and qRT-PCR relative gene expression data (right, Kruskal-Wallis rank sum test of *P* values followed by Benjamini-Hochberg adjustment of *P* values and Wilcoxon rank sum test pairwise comparison) of *BMP15* and *HIST1H1T*, respectively. Primordial (PrF); primary (PF); and secondary ovarian follicles (SF).

**Supplementary Table S1 Table of selected gene primer design for qRT-PCR.** The genes, *HIST1H1T* and *BMP15*, were investigated utilising β-actin (BACT) as a housekeeping gene.

**Supplementary Table S2 Functionally enriched GO and KO terms during primordial-to-primary ovarian follicle transition in domestic cat.** Enriched terms of interest (left) with respective gene symbols (right) from Metascape analysis (http://metascape.org).

**Supplementary Table S3 Functionally enriched GO and KO terms during the primary-to-secondary transition in domestic cat*.*** Enriched terms of interest (left) with respective gene symbols (right) from Metascape analysis (http://metascape.org).

**Supplementary Table S1**

| Gene | GenBank ID | Primer sequence 5’ – 3’ | T_A_ (°C) | | Product size (bp) |
| --- | --- | --- | --- | --- | --- |
| *HIST1H1T* | XM_003985721 | HST1H1T-q-fw: GGC TCC TTC AAG CTC AAC A  HST1H1T-q-rv: CCT TAG CCT TCC TGC TGC | | 58 | 202 |
| *BMP15* | NM_001165898 | BMP15-q-fw: GGA GTT GTA CCA GCG TTC A  BMP15-q-rv: GGA AAC GAG CTA GGT GGA | | 57 | 227 |
| *BACT* | AB051104 | qfw: GAG CAG GAG ATG GCC ACG  qrv: CTC GTG GAT GCC ACA GGA | | 62 | 159 |

**Supplementary Table S2**

| **Description** | **Gene symbols** |
| --- | --- |
| Extracellular structure organization | A2M,ABCA1,APOA1,APP,CAPN1,CAPN2,SERPINH1,CD34,CD44,COL1A1,COL1A2,COL3A1,COL4A1,COL4A2,COL4A5,COL5A2,COL6A1,COL6A2,COL6A3,COL11A1,COL15A1,CPB2,CST3,CTSL,CTSS,DAG1,DCN,ECM2,FBN1,FGFR4,GAS6,GPM6B,GSN,HPN,ITGA1,ITGA2,ITGA3,ITGA4,ITGB1,ITGB2,ITGB5,ITGB7,ITGB8,LAMA3,LAMB1,LAMB2,LAMC2,LOX,LRP1,MFAP2,MFAP4,MMP2,MMP7,MMP12,MYH11,NID1,TNFRSF11B,P4HA1,PLOD1,PLTP,PRKACB,QSOX1,SDC4,SDCBP,SPARC,TGFB1,TNFRSF1A,COL14A1,WT1,RECK,ITGA8,ADAM15,ABCG1,SPOCK2,FBLN5,PDPN,POSTN,SPINT2,EMILIN1,SULF1,ABCA5,ABI3BP,EFEMP2,F11R,JAM2,NTN4,RIC8A,NDNF,GFOD2,JAM3,LOXL3,MMP21,CCDC80,COL28A1,BMP4,CD151,CTSD,EFEMP1,PCOLCE,PRKCA,SDC2,TGFB3,LTBP4,CASK,ADAM9,P3H3,ADAMTS8,NCSTN,CHAD,EPYC,IGFALS,IGFBP2,IGFBP5,MATN2,MFGE8,MGP,NELL1,NELL2,PRELP,RELN,THBS3,THBS4,CCN4,EDIL3,FGL2,TINAG,FNDC8,TINAGL1,BMPER,OIT3,LGI3,GLDN |
| ECM-receptor interaction (Signalling by PDGF, Signalling by MET, MET activates PTK2 signalling, MET promotes cell motility, PI3K-Akt signalling pathway, PID AVB3 INTEGRIN PATHWAY, and collagen fibril organization) | CD44,CHAD,COL1A1,COL1A2,COL4A1,COL4A2,COL4A5,COL6A1,COL6A2,COL6A3,DAG1,HMMR,ITGA1,ITGA2,ITGA3,ITGA4,ITGB1,ITGB5,ITGB7,ITGB8,LAMA3,LAMB1,LAMB2,LAMC2,RELN,SDC4,THBS3,THBS4,ITGA8,SV2B,ACTB,ACTN4,AKT2,BAD,CCND1,BRAF,CAPN2,CAV1,ERBB2,FLNA,FLNB,FLT1,FYN,RAPGEF1,ARHGAP35,MYLK,PDGFRB,PIK3CD,PRKCA,TLN1,MYL9,PDGFC,MYL7,PDGFD,CD81,COL3A1,COL5A2,COL11A1,FBN1,NID1,PLAUR,JAM2,SDC2,TGFB1,CASK,NTN4,CD151,COL15A1,CTSL,CTSS,LOX,MMP7,PCOLCE,COL14A1,LOXL3,SERPINH1,P4HA1,PLOD1,P3H3,COL28A1,ITGB2,F11R,JAM3,A2M,CAPN1,CTSD,DCN,MMP2,MMP12,ADAM15,ADAM9,ADAMTS8,NCSTN,APP,SPARC,TGFB3,SDCBP,HPSE,GRB7,STAT3,STAT5A,STAT6,EPS15,GAB1,HPN,SH3GL2,SH3GL3,SPINT2,SH3KBP1,CCNE1,CSF1R,FGF1,FGFR4,GHR,IFNAR1,IL2RG,TSC2,YWHAB,CCNE2,FGF20,PPP2R3C,RPTOR,EIF4E1B,MFGE8,EDIL3,EMILIN1,PRNP,PTPRA,RPS6KA5,SPTBN4,ATP1B2,CPA2,CPB2 |
| Transmembrane receptor protein tyrosine kinase signalling pathway | ACTB,ADORA1,AP2A2,AP2B1,AKT2,ALK,APOD,APP,AXL,CD63,CHRNA3,CISH,COL1A1,COL4A1,COL4A2,COL4A5,CSF1R,CYBA,DCN,EFNB1,EPHA4,EPS15,ERBB2,EFEMP1,FGF1,FGFR4,FLT1,FYN,GAB1,GALNT3,GHR,GNAI2,GRB7,GRB10,RAPGEF1,GTF2F2,HPN,IGFBP2,IGFBP5,ITGA1,LOX,LRP1,MMP2,NDN,NKX31,NTRK1,PDGFRB,PDK4,PLAUR,POLR2K,PRKCA,PRKCQ,LGMN,PTPRA,PTPRG,RARRES2,SDC2,SDCBP,SH3,GL2,SNCA,STAT3,STAT5A,STAT6,TDGF1,TGFB1,TSC2,WNT5A,XDH,MAPKAPK3,NR4A3,TRADD,NRP1,CPNE3,KALRN,NEURL1,RPS6KA5,SLC9A3R1,NRG2,SPRY1,BAIAP2,SLC9A6,EMILIN1,SIRT2,CYFIP1,SULF1,SIK2,NCSTN,CBLC,DSTYK,NGEF,STAP1,FGF20,MYOF,BLNK,SH3KBP1,TRAT1,PLCE1,ESRP1,BAIAP2L1,PDGFC,MYORG,ELMO2,FUZ,PDGFD,GKAP1,AFAP1L2,MVB12B,CCBE1,ATP6V0D2,ATP6V1C2,GFRAL,ADCYAP1R1,BRAF,CALM3,CAV1,CHEK1,COL1A2,COL3A1,COL5A2,COL6A1,COL6A2,COL6A3,COL11A1,GABRB2,GABRB3,ITGA2,ITGA3,ITGB1,LAMA3,LAMB1,LAMB2,LAMC2,PGR,PRKACB,MAPK13,PTPN6,RPS6KA2,SH3GL3,SPARC,THBS3,THBS4,YWHAB,SPINT2,WWOX |
| Cell morphogenesis involved in differentiation | ACTB,ACTN4,APBB1,APOA1,APP,AXL,BMPR1B,CDH4,CHRNA3,COL6A1,COL6A2,COL6A3,CLDN3,CRABP2,CRMP1,CSF1R,DAG1,DBN1,DCC,DLX5,EFNB1,EPHA4,ERBB2,FLNA,FLNB,FYN,GAB1,GRB7,GRB10,ARHGAP35,ITGA4,ITGB1,ITGB7,LAMB1,LAMB2,LAMC2,LRP1,MAP1A,MAP1B,MATN2,MYO7A,NDN,NFIB,NTRK1,PIK3CD,PLXNA2,PPP3CA,PRKCA,PRKCQ,RELN,PTCH1,PTN,PTPN6,PTPRA,NECTIN1,SDC2,SLC1A3,STC1,THY1,COL14A1,VLDLR,WNT5A,SEMA3B,NR4A3,PLA2G10,ITGA8,NRP1,KALRN,SEMA5A,RPS6KA5,SLC9A3R1,NRXN3,FEZ1,CUL7,PLPPR4,HDAC6,SEC24B,BAIAP2,SLC9A6,PDPN,POSTN,SPINT2,KIF3A,PLXND1,CYFIP1,NEDD4L,OBSL1,MACF1,PLXNB2,NGEF,SIN3A,AUTS2,SH3KBP1,NIN,DOCK10,LIMS2,DSCAML1,SPTBN4,NTN4,SEMA6D,SPG11,FERMT3,DTNBP1,LINGO1,UNC5D,RILPL2,PHACTR1,RILPL1,APOD,TSPO,GRN,PTPRF,STK24,TNFRSF21,CRTAC1,JAM3,CD44,ITGA1,ENPP2,SH3GL2,AURKA,PACSIN2,RIMS1,CNTNAP2,CERT1,ANXA1,BMP4,C5AR1,FGF1,FLT1,GAS6,SERPIND1,ITGA2,ITGB2,LOX,CXCL9,PDGFRB,PLAUR,LGMN,RARRES2,SLC12A2,TGFB1,THBS4,TSC2,COLEC10,SWAP70,STAP1,ARHGEF16,STK39,ELMO2,NUP85,HSD3B7,PDGFD,IL17RC,MOSPD2,ALK,BBS4,EPS8,RAPGEF1,ITGA3,P2RX7,SERPINF1,SERPINI1,TGFB3,FZD1,NEURL1,TENM1,ZMYND8,PLEK2,ZMYND10,STYXL1,FNBP1L,MNS1,NDNF,FUZ,ZNF804A,SAXO1,CAV1,PMP22,PRNP,PTPRG,VIM,RAB29,ULK4,TBC1D2,ODF2L,TBC1D17,TTC21B,EFHC2,ITM2C,RTN4IP1,CSMD3,SDK1,BIN1,NKX25,EYA1,SH3GL3,TCF4,ZEB1,HMG20B,HEY1,HEY2,FGF20,ESRP1,EIF4ENIF1,CTDSP1,TGIF2,GDPD5,KLHL1,ANXA7,RND3,HPN,MSN,S100A13,SPARC,DLC1,BAMBI,BRWD1,ARHGAP15,SH3D19,SLC26A5,BAD,PDE3A,SERPINE2,SMARCD3,XRCC2,PDE5A,SIRT2,TENM4,LMOD3,AP2A2,AP2B1,CLTB,COL3A1,COL4A1,COL4A2,COL4A5,COL5A2,MAGOH,MMP2,MYH11,NELL2,PRKACB,MAPK13,PSMB9,PSMD13,RPS6KA2,RRAS,SDCBP,TLN1,TUBB2A,MYL9,CAP1,NCSTN,RPL26L1,TUBB6,CLSTN2,GNAI1,GNAI2,SSH2 |
| Cellular response to growth factor stimulus (cellular response to transforming growth factor beta stimulus, transmembrane receptor protein serine/threonine kinase and transforming growth factor beta receptor signalling pathway) | AMHR2,ANXA1,APOA1,APP,BMP4,BMPR1B,CAD,CAT,CAV1,CD44,CD63,COL1A1,COL1A2,COL3A1,COL4A2,NKX25,DAB2,DCN,DLX5,ERBB2,FBN1,FGF1,FGFR4,FLT1,FYN,GAB1,GALNT3,GAS6,GCLM,GLG1,GRB10,RAPGEF1,GTF2F2,ITGA3,ITGB1,ITGB5,LOX,MAP1B,NDN,NTRK1,NUMA1,PDE3A,PDGFRB,POLR2K,LGMN,PTN,SDCBP,SH3GL2,SNCA,SPARC,ZEB1,TDGF1,TFAP2B,TGFB1,TGFB1I1,TGFB3,WNT5A,XDH,FZD1,LTBP4,ITGA8,CASK,ADAM9,NRP1,CPNE3,CLDN1,MTMR4,PDCD5,SLC33A1,BMP15,FEZ1,SPRY1,BAIAP2,SLC9A6,POSTN,SPINT2,EMILIN1,SIRT2,CYFIP1,SULF1,DSTYK,BAMBI,FGF20,MYOF,F11R,CTDSPL2,WWOX,ESRP1,APPL2,TMEM100,TWSG1,SMURF2,FBXL15,VEPH1,FUZ,PDGFD,WFIKKN2,CCBE1,BMPER,NRROS,ANXA3,MEIS2,HDAC6,TTK,VIM,TBX20,TGIF2,BTBD11 |

**Supplementary Table S3**

| **Description** | **Gene symbols** |
| --- | --- |
| Chemotaxis (vasculature development, blood vessel morphogenesis, angiogenesis, and blood vessel development) | ALCAM,MST1,PIK3CG,PLAU,PLD1,PRKCA,CCL24,SLIT3,VAV3,GPNMB,PLXND1,LRTM1,ADGRB3,COMP,FAP,NOS3,TMEM204,E2F8 |
| Negative regulation of reproductive process (regulation of reproductive process, female gamete generation, single fertilization, oogenesis) | ZP4,HORMAD1,GPR149,WEE2,AR,NOS3,PLCZ1 |
| Regulation of cell adhesion | LAG3,PIK3CG,PLAU,PRKCA,TNFSF9,VAV3,GPNMB,PLXND1,AP1AR,ZP4,EGFLAM,IFNL1,TCF7 |
| PID IL8 CXCR1 PATHWAY(Oxytocin, GnRH, Sphingolipid, Ca2+, Apelin, Ras, and Phospholipase D signalling pathways) | PIK3CG,PLD1,PRKCA,ELK1,NOS3,PLCB4,COMP,VAV3,SLC4A4,PLA2G12B,GNB3,CYP4X1,TCF7,AR,PTCH2 |
| Sex differentiation (developmental process involved in reproduction, regulation of systemic arterial blood pressure, reproductive structure development, reproductive system development, ovulation cycle process, circulatory system process, and endocrine process) | AR,CYP17A1,NOS3,REN,SLIT3,TCF7,GPR149,MME,E2F8,HORMAD1,WEE2,COMP,GNB3,NFE2,PIK3CG,KCNIP4 |
| Peptide hormone processing (regulation of hormone levels) | MME,FURIN,REN,CRYM,CYP17A1,GNB3,PRKCA,ANO1,C1QC,COMP,PLAU |
| Signalling by Receptor Tyrosine Kinases (transmembrane receptor protein tyrosine kinase and ErbB signalling pathways) | ATP6V1G2,ELK1,MST1,NOS3,FURIN,PRKCA,NRG2,VAV3,AR,FSTL4,SIK2,TMEM204,SHCBP1 |
| Regulation of anatomical structure size(extra-nuclear estrogen signalling) | COMP,GNB3,NOS3,CCL24,VAV3,FSTL4,PPP1R15A,AP1AR,ELK1 |
